# Oxidative Stress-Derived Mitochondrial Dysfunction in Chronic Obstructive Pulmonary Disease: A Concise Review

**DOI:** 10.1155/2021/6644002

**Published:** 2021-03-12

**Authors:** Mariana A. Antunes, Miquéias Lopes-Pacheco, Patricia R. M. Rocco

**Affiliations:** ^1^Laboratory of Pulmonary Investigation, Carlos Chagas Filho Institute of Biophysics, Federal University of Rio de Janeiro, Rio de Janeiro, RJ, Brazil; ^2^National Institute of Science and Technology for Regenerative Medicine, Rio de Janeiro, Brazil; ^3^Rio de Janeiro Innovation Network in Nanosystems for Health-NanoSAÚDE/FAPERJ, Rio de Janeiro, Rio de Janeiro, Brazil

## Abstract

Chronic obstructive pulmonary disease (COPD) is a progressive and disabling disorder marked by airflow limitation and extensive destruction of lung parenchyma. Cigarette smoke is the major risk factor for COPD development and has been associated with increased oxidant burden on multiple cell types in the lungs. Elevated levels of reactive oxygen species (ROS) may significantly affect expression of biological molecules, signaling pathways, and function of antioxidant defenses. Although inflammatory cells, such as neutrophils and macrophages, contribute to the release of large quantities of ROS, mitochondrial dysfunction plays a critical role in ROS production due to oxidative phosphorylation. Although mitochondria are dynamic organelles, excess oxidative stress is able to alter mitochondrial function, morphology, and RNA and protein content. Indeed, mitochondria may change their shape by undergoing fusion (regulated by mitofusin 1, mitofusin 2, and optic atrophy 1 proteins) and fission (regulated by dynamin-related protein 1), which are essential processes to maintain a healthy and functional mitochondrial network. Cigarette smoke can induce mitochondrial hyperfusion, thus reducing mitochondrial quality control and cellular stress resistance. Furthermore, diminished levels of enzymes involved in the mitophagy process, such as Parkin (a ubiquitin ligase E3) and the PTEN-induced putative kinase 1 (PINK1), are commonly observed in COPD and correlate directly with faulty removal of dysfunctional mitochondria and consequent cell senescence in this disorder. In this review, we highlight the main mechanisms for the regulation of mitochondrial quality and how they are affected by oxidative stress during COPD development and progression.

## 1. Introduction

Chronic obstructive pulmonary disease (COPD) is a slowly progressive pulmonary disorder that affects over 300 million people worldwide. The Global Initiative for Chronic Obstructive Lung Disease currently estimates that 3 million people die annually due to COPD, a proportion that is expected to rise over the following 40 years as smoking becomes more prevalent in developing countries and populations grow older in high-income countries. COPD is an avoidable, treatable disease, which is characterized by progressive airflow limitation that may be fully or partly irreversible [1]. Corticosteroids are considered the gold-standard therapy for COPD; they alleviate patients' symptoms and increase the interval between exacerbations, with a potential delay in the decline of lung function. However, corticosteroids do not prevent disease progression, and a portion of patients do not benefit from these therapies due to smoking-induced steroid resistance [2].

Airflow limitation is frequently associated with an augmented, chronic inflammatory response and tissue remodeling in both airways and lung parenchyma, and occurs due to exposure to noxious particles or gases. Furthermore, comorbid disorders and exacerbations are directly linked to COPD severity [1, 3]. Cigarette smoke is the predominant risk factor for COPD, as it contains a large amount of substances that stimulate the production of reactive oxygen species (ROS), thus inducing an oxidant burden [4, 5]. In nonsmokers, COPD results from a complex interplay between long-term exposure to noxious particles and gases (e.g., occupational exposures to vapors, gases, dusts, or fumes) and individual genetic susceptibility (e.g., *α*-1 antitrypsin deficiency) [6].

Oxidative stress originates from an imbalance between oxidants derived from ROS and endogenous antioxidant production. In COPD, endogenous and exogenous oxidants are related to disease development [7]. In the chemical process of oxidation, electrons are withdrawn from molecules; leaked electrons then lead to the synthesis of reactive free radicals. Environmental pollutants and inhaled toxic gases, including industrial pollution, car exhaust fumes, occupational exposure to dusts, and cigarette smoking, are major exogenous sources of ROS production [8].

Among the endogenous sources of ROS, mitochondria are one of the main components involved in oxidant production. Mitochondrial ROS may be derived from up to 12 potential sites associated with nutrient oxidation and the electron transport chain (ETC), divided into two subgroups: the reduced nicotinamide adenine dinucleotide/nicotinamide adenine dinucleotide (NADH/NAD^+^) isopotential group and the ubiquinol/reduced ubiquinone (UQH_2_/UQ) isopotential group. These different sites make variable contributions to the overall release of O_2_^−^/H_2_O_2_ depending on cell type and tissue, which seems to be related to substrate availability and mitochondrial bioenergetics (Table 1) [9, 10]. In the ETC, electrons are transported through four respiratory chain complexes: complex I (NADH-coenzyme Q reductase), complex II (succinate dehydrogenase), complex III (coenzyme Q-cytochrome c reductase), and complex IV (cytochrome c oxidase), finally reacting with molecular oxygen, resulting in superoxide (O_2_^−^) production. Energy produced while electrons are carried through the ETC is used to pump protons into the intermembrane space, creating an electrochemical proton gradient (Δ*ψ*m) that is used by ATP synthase to generate ATP. Some electrons leak into the matrix and react with molecular oxygen to generate O_2_^−^, at several different sites, such as the oxoacid dehydrogenase complexes (which feed electrons to NAD+), ETC complexes I and III, and dehydrogenases, including ETC complex II. Thereafter, O_2_^−^ is further converted into hydrogen peroxide (H_2_O_2_) by superoxide dismutase (SOD). In the mitochondria, H_2_O_2_ is buffered by glutathione (GSH), thioredoxin/peroxiredoxins, and catalase systems [10]. Hence, high concentrations of O_2_^−^ play a key role in excess ROS production.

## 2. Neutrophil-Derived ROS in COPD

The cell damage and death observed in COPD airways have been attributed to oxidative stress triggered after smoking or air pollution exposure [11]. In a proportion of smokers who develop COPD, smoking cessation does not halt the disease progression, suggesting that an endogenous source promotes self-perpetuation of inflammation and tissue injury in these susceptible individuals [12–14]. The continuous release of inflammatory mediators (e.g., leukotriene B4 and interleukin-8) induces the recruitment and activation of additional inflammatory cells (especially neutrophils) into the lungs, which release further proteases, free radicals, and cytokines that promote destruction of the lung parenchyma, loss of lung elasticity [15], and hypersecretion of mucus [16].

Neutrophils from COPD patients exhibit several abnormal features, including secretion of elevated levels of ROS [17–19], high speed and low migration accuracy [20], and high levels of activation and degranulation of surface markers (CD63 expression) [21]. Indeed, degranulation products (e.g., inflammatory cytokines, ROS, and neutrophil elastase) increase inflammatory signaling and protease load in the lung tissue of individuals with COPD. Cigarette smoke increases IL-8 production in lung epithelial cells, acting as a chemoattractant for neutrophils, which release neutrophil elastase and promote degradation of the extracellular matrix components of lung tissue [22]. Neutrophil elastase not only digests extracellular matrix protein components [23] but also contributes to the loss of bronchial epithelial cells [22] and mucus hypersecretion by goblet cells [24]. Although *α*1AT is a hepatic antielastase that is physiologically able to inhibit neutrophil elastase [25], excess ROS can promote oxidation of the methionine residue at position 358 of the *α*1AT polypeptide chain [26, 27], resulting in its inactivation and leading to lung inflammation and tissue destruction. Furthermore, neutrophil myeloperoxidase catalyzes the oxidation reaction of chloride ions (Cl^−^) by hydrogen peroxide to generate the anionic ROS hypochlorite or hypochlorous acid (HClO); in turn, HClO reacts with low-molecular-weight amines (e.g., nicotine) to produce chloramines, which lead to cellular protein damage [28].

## 3. Oxidative Mitochondrial Dysfunction in COPD

A reduction in mitochondrial function—characterized by lower mitochondrial membrane potential, changes in ETC complex activities, diminished ATP synthesis, inefficient Ca^2+^ buffering, increased ROS production, altered mitochondrial dynamics, or release of proapoptotic factors—is observed during aging and in the course of many chronic diseases, including COPD. At baseline, as a result of mitochondrial oxidative phosphorylation, cells produce ROS from a minimal level of residual, unreduced molecular oxygen [29]. However, in the presence of harmful stimuli, the amount of mitochondrial ROS (mtROS) increases substantially. Each puff of cigarette smoke carries ~10^17^ oxidant molecules [5], including ROS such as superoxide, hydrogen peroxide, and hydroxyl anion, which may cause extensive damage to DNA, proteins, lipids, and organelles such as mitochondria. There is also degradation of mitochondrial DNA (mtDNA), which is much more likely to suffer oxidative damage than nuclear DNA and lacks elaborate repair machinery [30, 31]. The lipophilic fraction of cigarette smoke (including polycyclic aromatic hydrocarbons, aldehydes, amines, heavy metals, and phenolic compounds) is responsible for a reduction in mitochondrial membrane potential and ATP production, as well as concomitant generation of mtROS [32]. Adverse effects of cigarette smoke on mitochondrial respiratory function have already been described in several cell types, including cardiomyocytes (decrease in maximum respiration) [33], lymphocytes (reduction in complex IV activity) [34], and monocytes (impairment of mitochondrial membrane potential and apoptosis) [35]. Similarly, cigarette smoke causes direct damage to airway epithelial cells, resulting in airway inflammation involving several immune cells (e.g., neutrophils, macrophages, and lymphocytes) [1, 36]. Stress induced-mtROS also stimulate the release of mitochondrial damage-associated molecular patterns (mt-DAMPs) into the cytosol or extracellular space [37, 38] and activate the innate immune responses in lung tissue via pattern recognition receptors (PRRs) on the plasma membrane of alveolar epithelial cells and macrophages, resulting in further production of inflammatory cytokines via a caspase 1-dependent mechanism and formation of the nucleotide-binding oligomerization domain like receptor (NLR) P3 inflammasome, which induces the secretion of multiple interleukins related to type-1 airway inflammation [39, 40].

Long-term exposure to cigarette smoke leads to dysfunctional, elongated mitochondria, with fragmentation and disruption of mitochondrial cristae in COPD bronchial epithelial cells, resulting in extensive apoptosis and cellular senescence [41]. Alteration of mitochondrial biogenesis and mitophagy in the skeletal muscle cells of COPD patients may also contribute to loss of muscle strength [42], playing a role in the reduction in exercise endurance. COPD patients may exhibit reduced mitochondrial biogenesis, dysfunctional oxidative phosphorylation, and augmented mtROS production and diminished mitochondrial uncoupling protein-3 in skeletal muscle and airway smooth muscle (ASM) cells [43, 44]. In addition to an increased amount of mtROS, ASM cells from COPD patients also exhibit reduced mitochondrial membrane potential, ATP content, and basal and maximum respiration levels, which are associated with increased levels of biomarkers of inflammation (e.g., interleukin-6 and -8) and cell proliferation (granulocyte-macrophage colony-stimulating factor) [42].

Short-term exposure to cigarette smoke has been demonstrated to upregulate the expression of complexes II, III, IV, and V and the activity of complexes II, IV, and V in pulmonary cell mitochondria, which are slightly reduced compared to control values after removing smoking stimuli. However, cigarette smoking irreversibly decreases the expression and activity of complex I after an 8-week exposure [45]. This reduction suggests a complex II-driven oxidative phosphorylation induced by cigarette smoke. Indeed, this metabolic profile is accompanied by a redox response, which encompasses changes in the cellular and mitochondrial levels of pyridine nucleotides (oxidized and reduced nicotinamide adenine dinucleotides in their unphosphorylated—NAD^+^ or NADH—and phosphorylated—NADP^+^ or NADPH—states) and glutathione. Cigarette smoke exposure also induces a highly reduced environment in the mitochondrion, which is related to increased expression of the mitochondrial NADPH-generating enzymes isocitrate dehydrogenase-2 (IDH2) and nicotinamide nucleotide transhydrogenase (NNT) [45]. Accordingly, Cormier et al. [46] demonstrated that nicotine—the primary addictive component of tobacco smoke—binds to ETC complex I and inhibits its NADH-ubiquinone reductase activity in brain mitochondria. Nicotine binding results in inhibition of electron flow from NADH to complex I [46], which is associated with a detectable decrease in oxygen consumption by mitochondria. Several studies have demonstrated that nicotine may affect other ETC complexes (II-IV) as well [47–49], but the potential inhibitory effects of nicotine on these other complexes have yet to be completely elucidated. Primary human lung epithelial cells (SAEC and NHBE) are also sensitive to cigarette smoke extract; 15-day treatment *in vitro* promoted a significant reduction in levels of ETC complexes I, II, III, and IV, while protein expression of complex V remained unaffected [50].

Mitochondria also seem to be an intracellular target for cadmium, a heavy metal that is found in tobacco smoke. In renal epithelium, cadmium directly compromised mitochondrial function by increasing mitochondrial permeability and swelling, thus inhibiting respiration and increasing ROS production [51]. Although the pathological mechanisms of cadmium toxicity in mitochondria have not been completely elucidated, it has been reported to accumulate intracellularly and affect both mitochondrial ETC and membrane permeability [52]. In addition, cadmium is a powerful uncoupling agent that inhibits succinate- and malate/pyruvate-stimulated respiration. Cadmium interacts with complex I at the ubiquinone and NADH sites, resulting in complex I inhibition in rat hepatic mitochondria. Finally, cadmium exposure inhibits complex IV (cytochrome c oxidase) by inhibiting ETC downstream ubiquinone [53].

### 3.1. Altered Mitochondrial Dynamics

Mitochondrial morphology is maintained within a fine balance between two opposite processes: fusion and fission [54, 55]. Proteins involved in the regulation of mitochondrial morphology and maintenance of the fission-fusion balance are necessary for overall cellular health. Mitochondrial fission is necessary to preserve an ideal number of mitochondria during cellular growth and division processes, while fusion enables the unification of mitochondrial compartments. Mitochondrial fusion has been reported as a strategy to protect cells against mitochondrial DNA (mtDNA) mutations by allowing functional complementation of mtDNA genes [56]. Elongated mitochondria are preserved from mitophagy, exhibit more cristae and augmented ATP synthase activity, and are able to ensure ATP production [57]. Nevertheless, prolonged permanence of mitochondria in the elongated state may contribute to higher intracellular ROS and lower mitochondrial respiration, leading to cellular senescence—a major factor in the pathogenesis cascade of COPD [58].

There are three large guanosine triphosphatases (GTPases) responsible for the mitochondrial fusion process: mitofusion (Mfn) 1 and Mfn2 (both in the mitochondrial outer membrane), and optic atrophy protein 1 (Opa1), in the mitochondrial inner membrane. Long-term exposure to cigarette smoke has been shown to induce morphologic changes in the mitochondrial structure of human bronchial epithelial cells, including fragmentation, branching, and altered number of cristae, accompanied by a significant increase in fission and fusion markers, oxidative phosphorylation proteins, and oxidative stress markers (e.g., manganese superoxide dismutase) [41]. In baseline conditions, Mfn2 helps to maintain mitochondrial networks with elongated branching, while fission proteins have a less prominent role. Following cigarette smoke exposure, Mfn2 expression and function are diminished in human airway smooth muscle cells, while an increase in expression of mitochondrial fission protein (dynamin-related protein 1, Drp1) is observed [59]. On the other hand, cigarette smoke has been shown to markedly increase expression of Mfn1, Mfn2, and Opa1 in airway epithelial cells [41]. Elongated mitochondria have also been observed in alveolar epithelial cells and lung fibroblasts [60, 61], accompanied by reduced ATP levels [60] and cellular senescence.

Exposure to cigarette smoke facilitates mitochondrial fission in human smooth muscle cells, which may contribute to cellular apoptosis and mitophagy in COPD [59]. The most relevant mediator in this process is the fission protein dynamin-related protein 1 (Drp1). Drp1 translocates from the cytosol to the outer mitochondrial matrix, where it interacts with the human fission protein-1, mitochondrial fission factor, and mitochondrial dynamics proteins of 49 kDa and 51 kDa, and oligomerizes to form a spiral that leads to constriction and breakage of mitochondrial membranes. Mitochondrial fission ensures equal division of mitochondrial numbers during cell division and mediates the selective removal of damaged mitochondria by mitophagy. Sundar et al. [50] showed that, *in vitro*, cigarette smoke extract-treated primary human lung epithelial cells developed an imbalance of proteins involved in mitochondrial fission (augmented Drp1) and fusion (diminished Mfn2). Additionally, Aravamudan et al. [59] found that cigarette smoking-induced mitochondrial ROS accelerates the course of phosphorylation of Drp1 in human airway smooth muscle cells, leading to mitochondrial fragmentation. Drp1 inhibition prevented the effects of cigarette smoke on mitochondrial morphology changes, which are mediated by multiple signaling and transcriptional pathways that are relevant to COPD pathogenesis. These include activation of extracellular signal-regulated kinase (ERK), phosphatidylinositol 3-kinase (PI3K)/protein kinase B (Akt), protein kinase C (PKC), and proteasome pathways, as well as transcriptional regulation via factors such as NF-*κ*B and nuclear erythroid 2-related factor 2 [59].

### 3.2. Impaired Mitophagy

**Figure 1:**
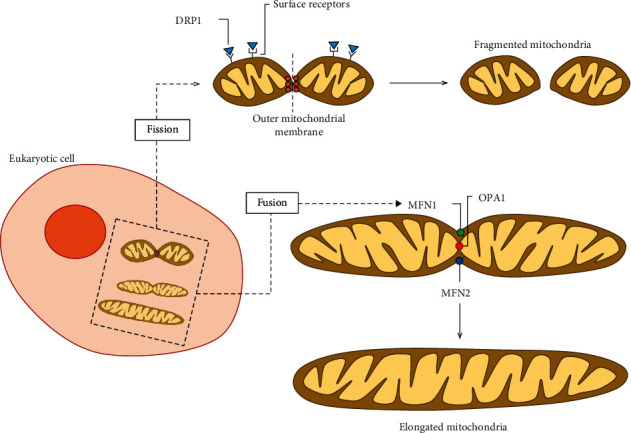
Mitochondrial dynamics in eukaryotic cells. Diverse processes contribute to generation and degradation of mitochondria within cells. During fission, recognition of activated dynamin-related protein 1 (DRP1) by surface receptors (fission protein 1, mitochondrial fission factor, mitochondrial dynamic protein 49, and mitochondrial dynamic protein 51) leads to fragmentation of the outer mitochondrial membrane (OMM) and formation of a new mitochondrion. Mitochondria may acquire an elongated design by fusion, where mitochondrial membranes tether through fusion proteins in the inner mitochondrial membrane (IMM), such as optic atrophy 1 (OPA1), and OMM, such as mitofusin 1 (MFN1) and MFN2.

Mitochondrial autophagy, known as mitophagy, plays a pivotal role in the removal of damaged mitochondria. However, several reports have demonstrated that cigarette smoke may lead to impaired mitophagy, resulting in accumulation of damaged mitochondria in the cytoplasm, where they are able to form a perinuclear cluster and induce damage to nuclear DNA (due to excess mtROS) and cellular senescence [60, 62]. Mitophagy is an important process to maintain a healthy intracellular environment by removing damaged or dysfunctional mitochondria [63–65]. The maintenance of the Δ*ψ*m is a *sine qua non* condition for mitochondrial ATP synthesis. Membrane depolarization below a certain Δ*ψ*m may signal dysfunctional mitochondria and is a prerequisite for mitophagy [66–68] (Figure 1). Although essential, mitochondrial depolarization alone does not initiate mitophagy, which only begins when mitochondria are depolarized and fail to regain their membrane potential to protect themselves from detrimental effects. This process engages the translocation of cytosolic proteins that tag mitochondria for mitophagy. These include an E3 ubiquitin ligase (Parkin) and the PTEN-induced putative kinase 1 (PINK1), an upstream regulator of Parkin [69, 70]. In the mitochondria, Parkin mediates degradation of Mfn2, which prevents mitochondrial fusion. During this process, damaged mitochondria are encompassed in microtubule-associated protein 1 light-chain 3 (LC3) mediated autophagosomes [71], which fuse with lysosomes to be sequentially eliminated from the cell [63–65, 71, 72].

**Figure 2:**
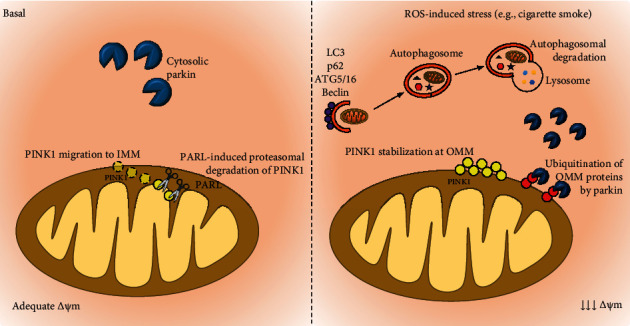
Mitophagy regulation. In healthy mitochondria, the kinase PINK1 is constitutively repressed by import into the inner mitochondrial membrane (IMM) and degradation by the rhomboid protease PARL. In uncoupled mitochondria, import of PINK1 into the IMM is avoided, so PINK1 is bypassed from PARL and accumulates in the outer mitochondrial membrane (OMM), acting as a signal to mark dysfunctional mitochondria for Parkin, which then conjugates ubiquitin (Ub) to several proteins on the OMM and mediates proteasomal degradation of mitofusins 1 and 2. Finally, Parkin induces engulfment of the damaged mitochondria by autophagy compartments. Several proteins, including autophagy-related genes (ATGs), beclin-1, microtubule-associated protein 1 light-chain 3 (LC3), and p62, facilitate the formation of autophagosomes.

In isolated mitochondria from human fetal lung fibroblasts (HLF), carbonyl cyanide m-chlorophenyl hydrazine (CCCP, a Parkin mitochondrial translocation agent) was used to induce mitochondrial depolarization and to test whether cigarette smoke could impair Parkin mitochondrial translocation (Figure 2). Ahmad et al. [60] reported that cigarette smoke extract not only leads to permanent mitochondrial dysfunction, inducing defective mitophagy by altering cytosolic Parkin levels and translocation in HLF, but also induces perinuclear mitochondrial accumulation, resulting in DNA damage and cellular senescence. Another cytosolic protein that can interact with Parkin, resulting in its inhibition, is p53. In one study, exposure to cigarette smoke for 15 days induced an increase in cytosolic levels of p53, resulting in its increased association with Parkin, reduced mitochondrial translocation of Parkin and, ultimately, impaired mitophagy [60].

**Figure 3:**
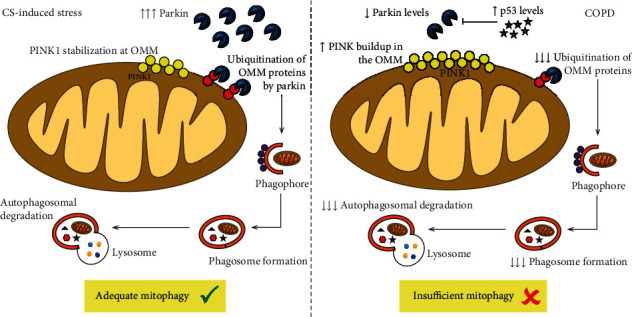
Impaired mitophagy in COPD. In regular conditions, cigarette smoke- (CS-) induced mitochondrial damage stabilizes PINK1 on the outer mitochondrial membrane (OMM), which signals for Parkin recruitment to the mitochondrion from an abundant cytosolic pool, leading to sufficient mitophagy, preventing the accumulation of dysfunctional mitochondria, and slowing cellular senescence. In COPD, decreased levels of cytosolic Parkin seem to be related to insufficient mitophagy, which increases the number of damaged mitochondria with stabilized PINK1, leading to further PRKN reduction by PINK1-mediated proteasomal degradation. In COPD, increased levels of p53, a cytosolic protein capable of inhibiting Parkin, may contribute to the reduced availability of Parkin molecules for proteasomal degradation of PINK1.

Both PINK1 and Parkin have been associated with the pathogenesis cascade of COPD with respect to the regulation of mitophagy. However, the existing data are still conflicting regarding the status of mitophagy and its role in disease progression. Previous studies reported elevated mitochondrial accumulation of PINK1 and reduction of Parkin expression in COPD lungs, but not in non-COPD smokers, suggesting that an increase in PINK1/Parkin ratio is not only dependent on smoking status but correlates with COPD pathogenesis (Figure 3) [41, 60, 62]. Airway epithelial cells from Parkin knockout mice exposed to cigarette smoke demonstrated accumulation of damaged mitochondria and increased mtROS accompanied by accelerated cellular senescence [73]. However, additional studies have found that mitophagy activation is not sufficient to decelerate cellular senescence in COPD pathogenesis [60, 62]. *In vitro* overexpression of Parkin, but not of PINK1, attenuated mtROS production and cellular senescence, suggesting that Parkin levels can be the rate-limiting factor in PINK1/Parkin-mediated mitophagy during cigarette smoke exposure [73]. Accordingly, Ito et al. demonstrated that cigarette smoke induces mitochondrial damage, increases mtROS production, and initiates senescence of primary human bronchial epithelial cells (HBECs), which may be reversed by inducing overexpression of Parkin in HBECs [62]. Conversely, Ahmad et al. suggested that Parkin overexpression can restore mitochondrial mass accumulation but does not have a significant impact on cellular senescence when cigarette smoke (15-day exposure) has already established cellular senescence [60].

Besides PINK1/Parkin, which are required for mitochondrial degradation via mitophagy, there are also proteins responsible for mitochondrial biogenesis and maintenance of cellular energetics, such as peroxisome proliferator-activated receptor *γ* coactivator 1*α* (PGC-1*α*), which has been implicated in mitochondrial dysfunction associated with chronic lung diseases. PGC-1*α* is an integrator of multiple signaling pathways and plays a central role in the regulation of mitochondrial biogenesis and oxidative stress by mitochondrial transcription factor A (TFAM), which ensures mitochondrial DNA replication during mitochondrial biogenesis. Konokhova et al. [74] demonstrated that transcript levels of regulators of mitochondrial biogenesis and oxidative metabolism are upregulated in COPD; however, single-fiber analyses of oxidative-deficient fibers (cytochrome c oxidase-deficient and succinate dehydrogenase-positive fibers) and normal fibers revealed an impairment in mitochondrial biogenesis in COPD. While healthy individuals exhibit an increase in mtDNA and TFAM protein in oxidative-deficient muscle fibers compared to regular muscle fibers, which suggests a compensatory attempt to increase energy levels in oxidative-deficient cells, COPD patients present similar levels in both oxidative-deficient and regular muscle fibers. Such findings indicate that, although there is an increase in signaling factors for mitochondrial biogenesis in the muscle of patients with COPD, impairment in the translation of such signals precludes the recovery of normal oxidative capacity [74].

## 4. Antioxidant Defenses in COPD

**Figure 4:**
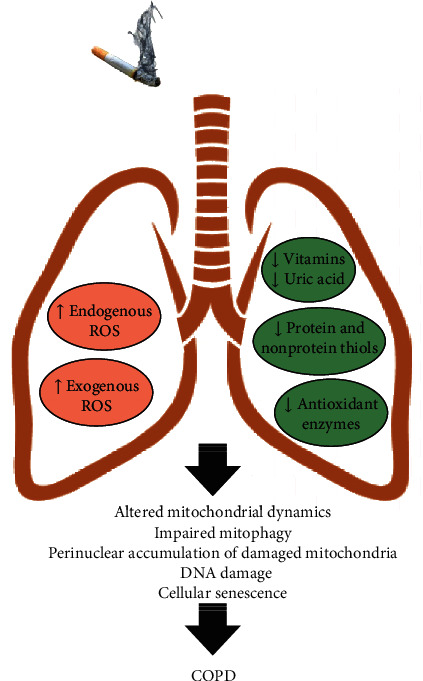
Role of oxidative stress in COPD pathogenesis. Cigarette smoke-induced oxidant burden activates intracellular signaling pathways that result in oxidant-antioxidant imbalance, leading to harmful lung cellular damage. The reduction of pulmonary antioxidant defenses induces changes in mitochondrial dynamics (e.g., elongated mitochondria), impaired clearance of damaged mitochondria (mitophagy), accumulation of dysfunctional mitochondria in the perinuclear region of lung cells, DNA damage, and subsequent cell senescence during COPD pathogenesis.

A series of antioxidant defenses, such as reduced GSH, has been developed by the lungs as the respiratory tract is permanently exposed to external and endogenous oxidative stress. A significant portion of the total GSH produced within the mitochondria is intended to balance endogenous production of ROS as a metabolism byproduct [75]. The enzyme glutamate-cysteine ligase is responsible for regulating the synthesis of GSH [76]. Nevertheless, bronchial epithelial cells and alveolar macrophages of smokers and patients with COPD have demonstrated low levels of GSH [77] and other GSH-dependent antioxidant enzymes (e.g., class-pi glutathione S-transferase, glutathione S-transferase mu 1, and glutathione peroxidase) [78]. The oxidative stress state of COPD induces the occurrence of critical events, such as epithelial permeability, lipid peroxidation, and cellular antioxidant GSH depletion in the alveolar epithelium, leading to apoptosis and inflammation characteristics in the pathological cascade of this disorder. As a protective mechanism against environmental oxidants, the lung epithelial lining fluid contains several antioxidants, including ascorbic acid (vitamin C), *α*-tocopherol (vitamin E), and uric acid. Levels of these antioxidants in the lungs appear to correlate inversely with pulmonary dysfunction in COPD (Figure 4). In addition, long-term antioxidant supplementation has been shown to minimize the risk of chronic lung disease development by 10%, as well as to reduce pulmonary carbonyl stress [79, 80]. Lung tissue from COPD patients often exhibits elevated expression of transforming growth factor-*β* (TGF-*β*), which can inhibit expression of several antioxidant enzymes in airway smooth muscle cells, including catalase and superoxide dismutase 2 (SOD2) [81]. These enzymes are regulated by the transcription factor FOXO3 and play a key role in maintaining a balance in mtROS production. Deficient FOXO3 activity has been reported in association with cigarette smoke-induced inflammation and airspace enlargement in COPD [82]. Furthermore, recent evidence demonstrated that serum antioxidant levels from smokers without COPD are significantly increased compared to those of smokers with COPD, suggesting that low antioxidant defenses may be related to COPD onset and progression [73]. Nevertheless, further studies are needed to clarify these differences, as no correlation was found between spirometric data and antioxidant levels in healthy nonsmokers, smokers, and COPD patients [83–86].

## 5. Conclusion

In this concise review, we have provided an overview of some key endogenous sources of oxidative burden and their role in COPD pathogenesis. The oxidative/antioxidative balance is finely maintained by a functional antioxidant system (e.g., GSH, ascorbic acid), aimed at neutralizing ROS production by inflammatory cells (particularly neutrophils) and mitochondria. Prolonged exposure to cigarette smoke induces massive infiltration of neutrophils and an imbalance in mitochondrial function, leading to excessive production of ROS, altered mitophagy, and perinuclear accumulation of defective mitochondria, which result in DNA damage and cellular senescence in COPD. Conversely, antioxidant levels are drastically reduced in COPD, predisposing to tissue oxidative damage. Although oxidative stress has been correlated with pulmonary and systemic features of COPD, the evidence is still controversial and insufficient to demonstrate whether boosting antioxidant mediators might indeed reverse cellular senescence. Considering the impact of oxidative load on the lives of patients with COPD, antioxidant-target therapies should be further studied with a view to identifying therapeutic strategies with potential impact on quality of life and life expectancy.

## Figures and Tables

**Table 1:** Mitochondrial sources of ROS production.

Isopotential group	Enzyme	Site of production	Tissue/organ
NADH/NAD^+^	*α*-Ketoglutarate dehydrogenase (KDGH)	FAD	Liver, skeletal muscle
	Pyruvate dehydrogenase (PDH)	FAD	Liver, skeletal muscle
	Branched-chain keto acid dehydrogenase	FAD	Skeletal muscle
	2-Oxoadipate dehydrogenase	FAD	Skeletal muscle
	Complex I	FMN	Cardiac muscle
UQH_2_/UQ	Complex I	UQ binding site	Skeletal muscle
	Complex II	FAD	Skeletal muscle, cardiac muscle, liver
	Complex III	UQ binding site	Skeletal muscle, cardiac muscle, liver
	Electron transfer flavoprotein: ubiquinone oxidoreductase	FAD	Undetermined
	*sn*-Glycerol-3-phosphate dehydrogenase (3GPDH)	FAD	Skeletal muscle, cardiac muscle, liver
	Proline dehydrogenase	FAD	Undetermined
	Dihydroorotate dehydrogenase	FAD	Undetermined

NADH/NAD^+^: reduced nicotinamide adenine dinucleotide/nicotinamide adenine dinucleotide isopotential group; UQH_2_/UQ: ubiquinol/reduced ubiquinone isopotential group; FAD: flavin adenine dinucleotide.

## Data Availability

No data were used to support this study.
